# Validation of the hepatocellular carcinoma early detection screening algorithm Doylestown and aMAP in a cohort of Chinese with cirrhosis

**DOI:** 10.1002/jcla.24296

**Published:** 2022-02-26

**Authors:** Yunsong Qian, Linhong Li, Li Ma, Rengbin Ji, Sheng Ying, Juhong Zhou, Liyun Fu, Gang Yang

**Affiliations:** ^1^ Department of Infection and Hepatology Hwa Mei Hospital, University of Chinese Academy of Sciences Ningbo, Zhejiang China; ^2^ Research and Development Division Oriomics Biotech Hangzhou, Zhejiang China; ^3^ Key Laboratory of Diagnosis and Treatment of Digestive System Tumors of Ningbo Zhejiang China

**Keywords:** alpha‐fetoprotein, aMAP score, Doylestown algorithm, early screening, hepatocellular carcinoma

## Abstract

**Background:**

Previous studies have developed some blood‐based biomarker algorithms such as the Doylestown algorithm and aMAP score to improve the detection of Hepatocellular carcinoma (HCC). However, no one has studied the application of the Doylestown algorithm in the Chinese. Meanwhile, which of these two screening models is more suitable for people with liver cirrhosis remains to be investigated.

**Methods:**

In this study, HCC surveillance was performed by radiographic imaging and testing for tumor markers every 6 months from August 21, 2018, to January 12, 2021. We conducted a retrospective study of 742 liver cirrhosis patients, and among them, 20 developed HCC during follow‐up. Samples from these patients at three follow‐up time points were tested to evaluate alpha‐fetoprotein (AFP), the Doylestown algorithm, and aMAP score.

**Results:**

Overall, 521 liver cirrhosis patients underwent semiannual longitudinal follow‐up three times. Five patients were diagnosed with HCC within 0–6 months of the third follow‐up. We found that for these liver cirrhosis patients, the Doylestown algorithm had the highest accuracy for HCC detection, with areas under the receiver operating characteristic curve (AUCs) of 0.763, 0.801, and 0.867 for follow‐ups 1–3, respectively. Compared with AFP at 20 ng/ml, the Doylestown algorithm increased biomarker performance by 7.4%, 21%, and 13% for follow‐ups 1–3, respectively.

**Conclusions:**

Our findings show that the Doylestown algorithm performance appeared to be optimal for HCC early screening in the Chinese cirrhotic population when compared with the aMAP score and AFP at 20 ng/ml.

## Graphical abstract

In this study, HCC surveillance was performed by radiographic imaging and testing for tumor markers every 6 months from August 21, 2018, to January 12, 2021. We conducted a retrospective study of 742 liver cirrhosis patients. Samples from these patients at three follow‐up time points were tested to evaluate alpha‐fetoprotein (AFP), the Doylestown algorithm, and aMAP score. Overall, 521 liver cirrhosis patients underwent semiannual longitudinal follow‐up three times. Five patients were diagnosed with HCC within 0–6 months of the third follow‐up. We found that for these liver cirrhosis patients, the Doylestown algorithm had the highest accuracy for HCC detection, with areas under the receiver operating characteristic curve (AUC) of 0.763, 0.801, and 0.867 for follow‐ups 1–3, respectively. Compared with AFP at 20 ng/ml, the Doylestown algorithm increased biomarker performance by 7.4%, 21%, and 13% for follow‐ups 1–3, respectively. Our findings show that the Doylestown algorithm performance appeared to be optimal for HCC early screening in the Chinese cirrhotic population when compared to the aMAP score and AFP at 20 ng/ml.

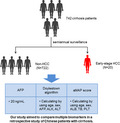

## 1 INTRODUCTION

Hepatocellular carcinoma (HCC) is the fifth most common cancer and the second leading cause of cancer death in China.^1^, ^2^ Meanwhile, the prognosis of HCC patients remains poor. Patients with advanced HCC have few treatment options, with 5‐year survival rates ranging from 5% to 14%, whereas patients with early HCC can undergo radical treatments, including surgical resection, ablation, and liver transplantation.^3^, ^4^ The 5‐year survival rate for HCC patients diagnosed at an early stage is up to 75%.^5^, ^6^, ^7^ Therefore, early detection of HCC is a key component toward reducing HCC mortality.

About 80%–90% of HCC cases occur in patients with cirrhosis and cirrhosis is also the major cause of liver disease‐related morbidity and mortality worldwide.^6^, ^8^ Chinese guideline for stratified screening and surveillance of primary liver cancer (2020 Edition) recommends abdominal ultrasonography combined with serum a‐fetoprotein (AFP) every 6 months as a surveillance program for HCC in patients with cirrhosis.^9^ However, ultrasound is dependent on operator experience and difficult to perform in obese patients.^10^, ^11^ AFP has suboptimal sensitivity and specificity (41% to 65% and 80% to 94%), and its usefulness has been widely debated.^12^ At the currently used cutoff (20 μg/L), this strategy misses about a third of HCC in the early stage.^13^, ^14^ Novel surveillance strategies are urgently needed to improve the accuracy of early HCC detection.

Wang et al.^15^ developed a logistic regression algorithm named the Doylestown algorithm that utilizes AFP, age, gender, alkaline phosphatase (ALK), and alanine aminotransferase (ALT) levels to improve the detection of HCC, particularly for those with cirrhosis. However, it is uncertain whether the Doylestown algorithm is applicable for predicting HCC occurrence in the Chinese population. Recently, aMAP risk score consisting of age, gender, total bilirubin (TB), albumin (ALB), and platelets (PLT) was reported to predict HCC development in patients with chronic hepatitis.^16^ aMAP score has the advantage of assessing HCC risk with different ethnicities including Asian and Caucasian ethnicities. However, the cohort of this study is mainly hepatitis B, hepatitis C, and other chronic hepatitis patients. Whether aMAP score is suitable for assessing the risk of HCC in patients with cirrhosis is still uncertain. This study aims to compare multiple biomarkers (including AFP, the Doylestown algorithm, and aMAP score) in Chinese patients with cirrhosis and investigate the clinical utility of the Doylestown algorithm and aMAP score.

## 2 MATERIALS AND METHODS

### 2.1 Study populations

A total of 814 patients with liver disease from the Hwa Mei Hospital, University of Chinese Academy of Science were enrolled into our study between August 2018 and May 2020. The study was approved by the Ethics Committees of Hwa Mei Hospital (IRB No: PJ‐NBEY‐KY‐2018–023–01), and written informed consent was obtained from all participants. Serum and plasma were collected at each visit and stored at −80°C. Patients were followed with semiannual surveillance until HCC occurred, death, or study termination. HCC free status at the start of all patients was confirmed by radiological imaging, including computed tomography (CT) and magnetic resonance imaging (MRI). Patients with non‐cirrhotic, suspicious liver masses, or HCC before study enrollment were excluded. In addition, only those patients who had the required components of the Doylestown algorithm (age, gender, ALT, ALK, and AFP) and aMAP score (age, gender, TB, ALB, and PLT) were utilized in this study. A total of 72 cases were excluded for the following reasons: one case transfer to other hospitals for treatment, four non‐cirrhotic cases, and 67 cases with insufficient clinical data for analysis. Ultimately, 742 patients were enrolled for analysis. The racial background of all patients was uniformly Chinese. Summary etiologic data of the cohort are given in Table 1. Patients with hepatitis B or C were treated with nucleos(t)ide analogs or direct‐acting antiviral agent treatment, respectively.

**TABLE 1:** Etiologic data among 742 cirrhosis patients

Etiology	Number (%)
HBV^a^	517 (69.7)
HBV+ NAFLD^b^	79 (10.6)
HBV+Alcohol	27 (3.6)
HCV^c^	7 (0.9)
HCV+NAFLD	2 (0.3)
Alcohol	36 (4.9)
NAFLD	16 (2.2)
Cryptogenic	58 (7.8)

^a^ Hepatitis B virus.

^b^ Non‐alcoholic fatty liver disease.

^c^ Hepatitis C virus.

Patients were followed up from August 21, 2018, to January 12, 2021. During this period, HCC surveillance was performed by radiographic imaging (CT and/or MRI) and testing for tumor markers every 6 months. The diagnosis of HCC was made based on radiological imaging. This cohort has been followed for a median of 378 days (range of 254–840). 20 patients were eventually diagnosed with early HCC in this study (Table S1). Early‐stage HCC was defined as Barcelona Clinic Liver Cancer(BCLC) stage 0 or A.^17^

All laboratory data, including serum AFP and values for ALT, ALK, TB, ALB, and PLT were determined using standard methods at Hwa Mei Hospital clinical laboratory. More details for patient enrollment can be found in Supporting information.

### 2.2 Statistical methods

The Doylestown algorithm was calculated based on a previous study,^15^ as follows:

1/(1+EXP(‐(−10.307+(0.097*age[year])+1.645*Gender[male:1,female:0]+(2.314*log_10_AFP[ng/ml])+(0.011*ALK[U/L])+(−0.008* ALT[U/L])))).

The output value ranged from 0 to 1. The cutoff value of 0.5 was used to identify patients with HCC.

The aMAP score was calculated based on a previous study,^16^ as follows:

((age[year]*0.06+gender*0.89[male:1,female:0]+0.48*((log_10_bilirubin[μmol/L]*0.66) + (albumin [g/L]*‐0.085)) ‐ 0.01*platelets [10^3^/mm]) +7.4) / 14.77*100.

The output value ranged from 0 to 100. The cutoff value of 50 was associated with a medium‐risk group. The cutoff value of 60 resulted in a high‐risk group.

A *t* test was performed to compare AFP, Doylestown algorithm, and aMAP score. Moreover, their correlation was tested using Pearson's correlation coefficient. All statistical tests were two‐sided and evaluated at the 0.05 level of statistical significance. All statistical analyses were performed using the R language, version 3.6.0.

## 3 RESULTS

### 3.1 Distribution of AFP, Doylestown algorithm score (DAs), and aMAP score

Patients were followed up three times from August 21, 2018, to January 12, 2021. Among 742 patients with cirrhosis during that period, we conducted a retrospective study of 20 HCC cases and 722 cirrhosis control subjects. In the cirrhosis control group, 516 patients were followed up three times, 603 were followed up at least twice, and 722 patients were followed up at least once. Among the HCC cases, five patients were followed up three times at HCC diagnosis, 17 were followed up at least twice before diagnosis, and 20 patients were followed up at least once before diagnosis.

A total of 521 patients were followed up three times. As shown in Table 2, the mean age of these patients was 52 (range, 31–75) years. The majority of the patients were men (70%). The mean values for ALT, ALK, TB, ALB, and PLT are listed in Table 2. As Table 2 shows, TB, ALB, and PLT values remained similar over about 12 months for HCC and non‐HCC patients. Since these three factors are key components of the aMAP score, the aMAP score values were also similar over the three time points. The ALT and AFP levels were higher at the first follow‐up visit (Time 1) in non‐HCC patients, and the Doylestown algorithm values were also higher at Time 1. AFP levels of HCC patients increased close to the time of HCC diagnosis, and their Doylestown algorithm score (DAs) had the same trend.

**TABLE 2:** Individual component and the AFP, Doylestown algorithm, and aMAP score at different time points in patients with liver cirrhosis (*n* = 521)^a^

	Individuals followed for about 12 months with liver cirrhosis. Time, in months from the first collection to the last collection							
	Non‐HCC (*n* = 516)				HCC (*n* = 5)			
Analyte	Time 1^b^	Time 2^c^	Time 3^d^	*p* value^e^	Time 1	Time 2	Time 3	*p* value
ALT(U/L)^f^	32	27	28	2.28E−04	38	22	23	0.015
ALK(U/L)^g^	83	86	86	3.32E−11	110	102	99	0.449
TB(umol/L)^h^	17.0	17.0	17.4	0.049	13.5	15.3	14.1	0.041
ALB(g/L)^i^	43	44	46	1.43E−38	43	44	45	0.015
PLT(10^3^/mm)^j^	110	113	118	8.95E−18	119	114	128	0.247
AFP(ng/ml)^k^	9.7	4.1	3.6	4.59E−09	5.8	6.8	7.3	0.449
DA^l^	0.32	0.18	0.18	3.49E−04	0.44	0.50	0.53	0.247
aMAP^m^	60	60	60	5.35E−15	63	63	62	0.165

^a^ A total of 1563 samples consisting of three time points each from 521 individual patients were examined.

^b^ Time 1 is 12–18 months prior.

^c^ Time 2 is 6–12 months prior.

^d^ Time 3 is 0–6 months prior.

^e^ Friedman test.

^f^ Mean level of alanine aminotransferase (ALT) with the range indicated (in U/L).

^g^ Mean level of alkaline phosphatase (ALK) with the range indicated (in U/L).

^h^ Mean level of total bilirubin (TB) with the range indicated (in μmol/L).

^i^ Mean level of albumin (ALB) with the range indicated (in g/L).

^j^ Mean level of platelets (PLT) with the range indicated (in 103/mm).

^k^ Mean level of alpha‐fetoprotein (AFP) with the range indicated (in ng/ml).

^l^ Mean level of the Doylestown algorithm with the range indicated.

^m^ Mean level of the aMAP score with the range indicated.

**FIGURE 1:**
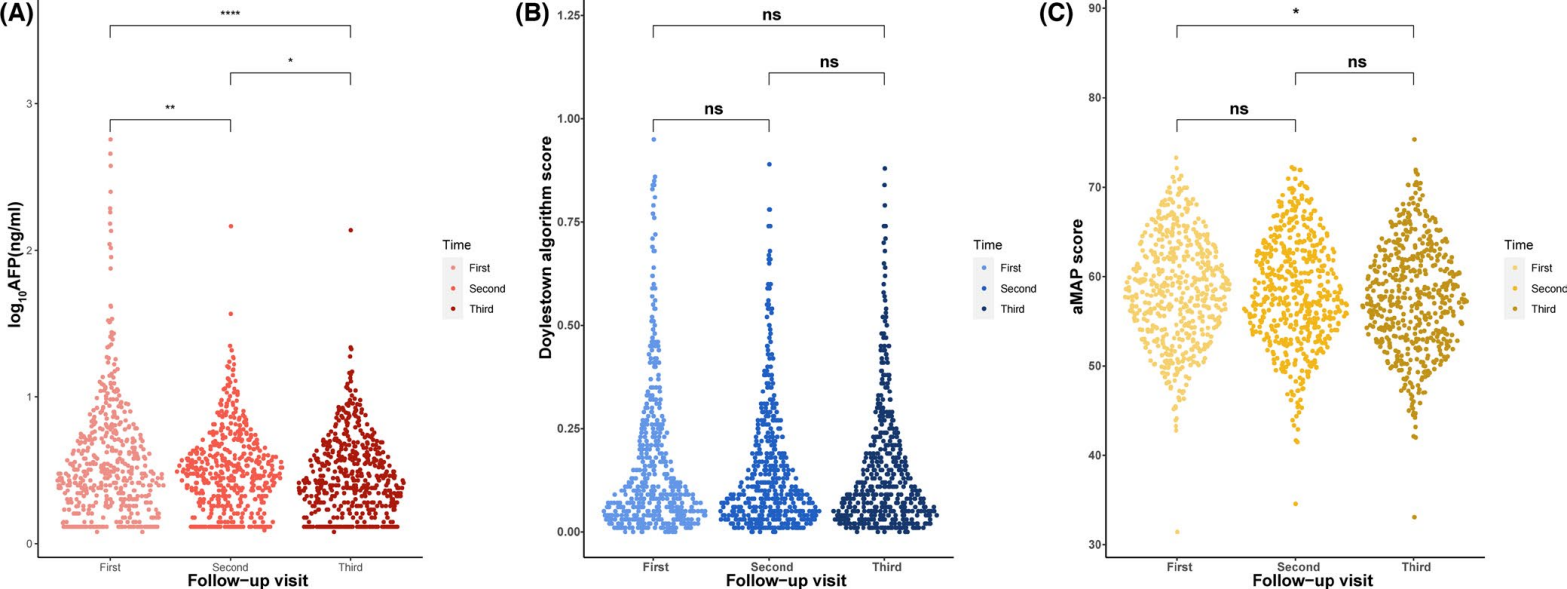
Distribution of AFP, Doylestown algorithm score (DAs), and aMAP score. Retrospective longitudinal data at three follow‐up visits were available from 516 patients without HCC and 5 patients with HCC. * Represents *p* < 0.05; ** represents *p* < 0.01; **** represents *p* < 0.0001; ns represents no significant difference

Longitudinal data were available from 521 patients at the first follow‐up visit (12–18 months before diagnosis), second follow‐up visit (6–12 months before diagnosis), and third follow‐up visit (0–6 months before diagnosis). Figure 1 shows the distribution of AFP, Doylestown algorithm score, and aMAP score in all patients at different times, respectively.

### 3.2 HCC Patient characteristics

The HCC patient characteristics are detailed in Table S1. All HCC patients had BCLC stage 0/A (0/11, A/9). Most HCC patients were diagnosed using MRI, except for two patients by CT. The maximum diameter of the tumor at the time of diagnosis was no more than 40mm (7–32 mm). Half of the HCC patients had undergone surgery after diagnosis. Among the HCC cases, 20 patients underwent biomarker assessment at 0–6 months before HCC diagnosis, 17 patients within 6–12 months before diagnosis, and 5 patients at 12–18 months before diagnosis.

### 3.3 Biomarker performance at three follow‐up visits before HCC diagnosis

**FIGURE 2:**
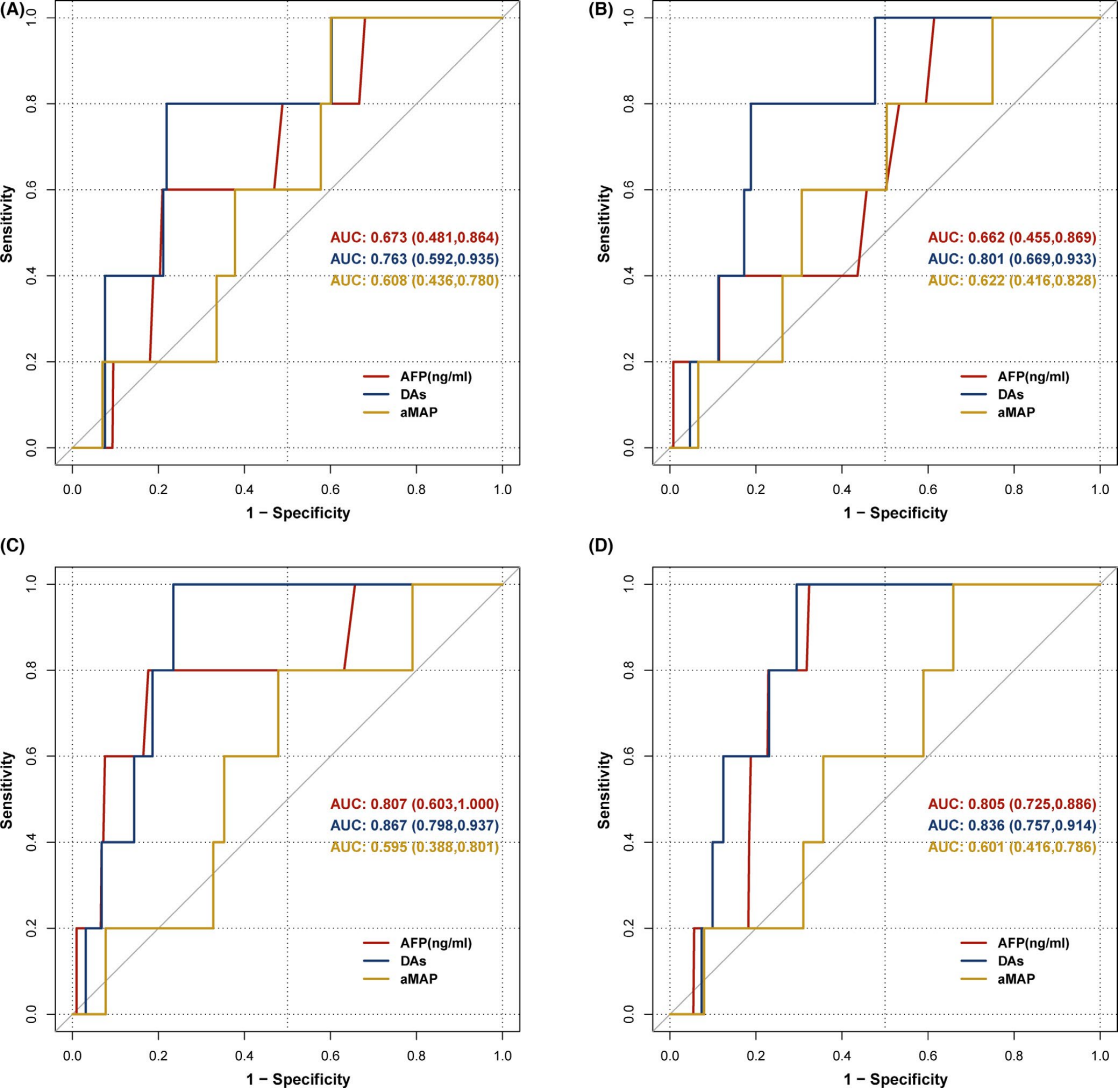
Receiver operating characteristic curves for patients with cirrhosis at three follow‐up visits before HCC diagnosis. (A) The first follow‐up visit. (B) The second follow‐up visit. (C) The third follow‐up visit. (D) The highest clinical factors value was recorded in the three follow‐up visits

A total of 521 liver cirrhosis patients underwent three longitudinal follow‐ups. Five patients were diagnosed with HCC within 0–6 months of the third follow‐up. The Doylestown algorithm and aMAP score values were calculated using patient clinical data. As for five HCC patients with three follow‐up visits, we found that the Doylestown algorithm provided the highest positive predictive value, the aMAP score had the highest sensitivity and negative predictive value, and the AFP model had the highest specificity (Table 3). The Doylestown algorithm demonstrated the highest accuracy for HCC detection, with an area under the receiver operating characteristic curve (AUC) of 0.763 12–18 months before diagnosis (95% CI, 0.592–0.935), 0.801 6–12 months before diagnosis (95% CI, 0.669–0.933), and 0.867 0–6 months before diagnosis (95% CI, 0.798–0.937) (Figure 2A–C). Furthermore, the diagnostic value of the Doylestown algorithm showed an increasing trend with the prolonged follow‐up time. The Doylestown algorithm and AFP demonstrated the best performance at 0–6 months before diagnosis, but the aMAP score had the best performance at 6–12 months before diagnosis, and the aMAP score showed a poor performance overall.

**TABLE 3:** Prediction accuracy for hepatocellular carcinoma development in patients with liver cirrhosis cohorts (*n* = 521) using different biomarkers

Follow‐up	First (12–18 months before diagnosis)				Second (6–12 months before diagnosis)				Third (0–6 months before diagnosis)			
	AFP−20^a^	DAs−0.5^b^	aMAP−50^c^	aMAP−60^d^	AFP−20	DAs−0.5	aMAP−50	aMAP−60	AFP−20	DAs−0.5	aMAP−50	aMAP−60
Sensitivity	0.00%	20.00%	100.00%	60.00%	0.00%	20.00%	100.00%	60.00%	0.00%	0.00%	100.00%	60.00%
Specificity	99.42%	96.32%	9.88%	64.34%	99.22%	94.96%	8.33%	61.43%	94.96%	93.41%	7.17%	61.43%
PPV	0.00%	5.00%	1.06%	1.60%	0.00%	3.70%	1.05%	1.49%	0.00%	0.00%	1.03%	1.49%
NPV	99.03%	99.20%	100.00%	99.40%	99.03%	99.19%	100.00%	99.37%	98.99%	98.97%	100.00%	99.37%

Abbreviations: NPV, Negative predictive value; PPV, Positive predictive value.

^a^ AFP‐20: Using the AFP cutoff values of 20 ng/ml.

^b^ DAs‐0.5: Using the Doylestown algorithm cutoff values of 0.5.

^c^ aMAP‐50: Using the aMAP score cutoff values of 50.

^d^ aMAP‐60: Using the aMAP score cutoff values of 60.

Next, we used the maximum values of ALT, ALK, AFP, TB, ALB, and PLT among three follow‐up visits of each patient for model construction, in order to explore whether this analytic strategy will further improve model performance. However, the new AUC generated by the new method (Figure 2D) was similar to the AUC calculated using the values of these laboratory variables at the third follow‐up visit (Figure 2C). Therefore, this new analytic strategy did not improve the model performance.

### 3.4 Biomarker performance at each follow‐up

**FIGURE 3:**
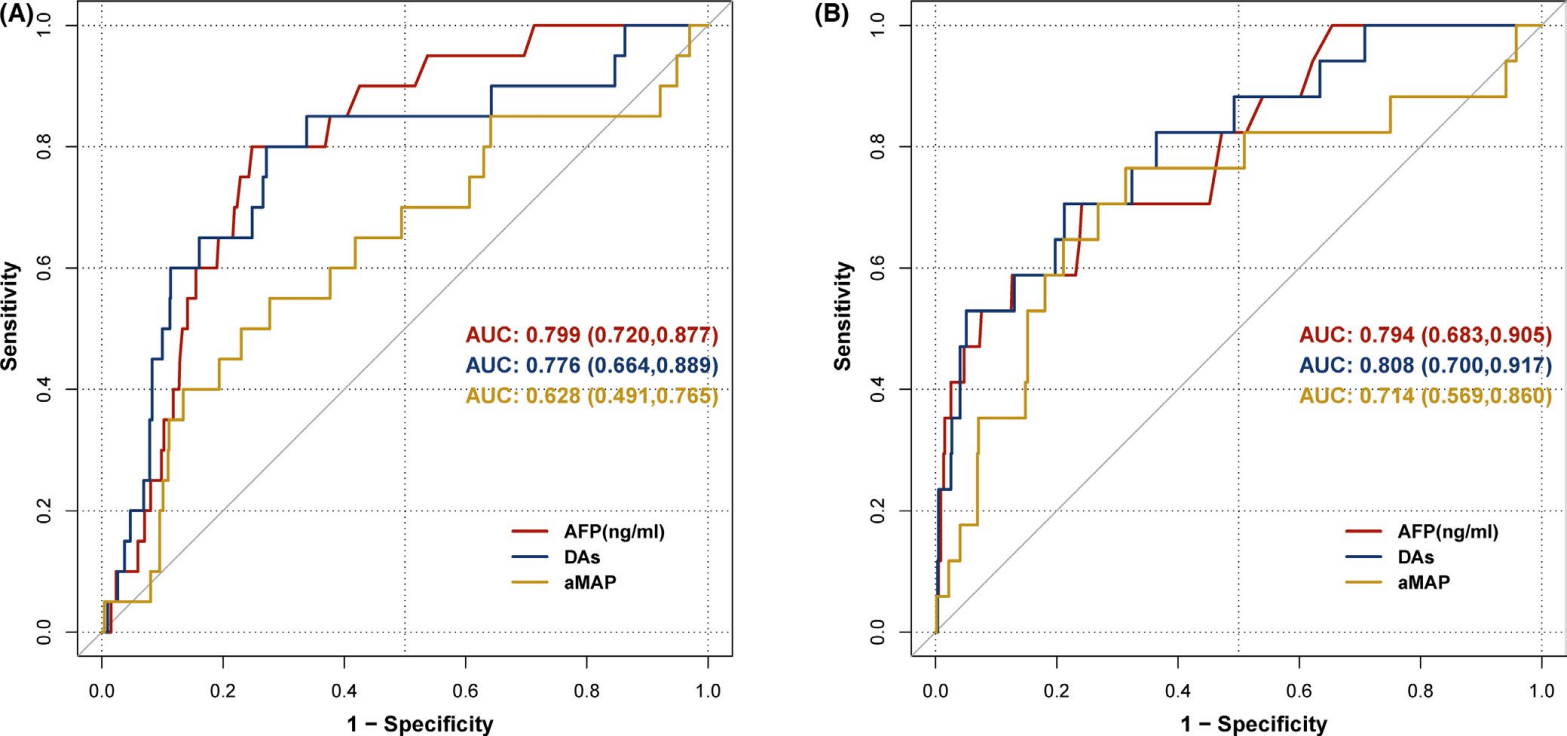
Receiver operating characteristic curves for patients with cirrhosis at each follow‐up. (A) The first follow‐up visit. (B) The second follow‐up visit

For very few HCC patients who were diagnosed at the third follow‐up in our cohort, we further analyzed the predictive value for all the cases with final HCC diagnosis with the data of cirrhosis patients at each follow‐up time. There were 742 liver cirrhosis patients with the data of the first follow‐up visit, and in which 20 HCC were diagnosed in the end. And a total of 610 liver cirrhosis patients were followed up twice with 17 HCC diagnoses at last. When adjusting the strategy to analyze all patients with liver cancer and cirrhosis at each follow‐up (Figure 3A, B), the AUC for the Doylestown algorithm increased from 0.763 to 0.776 at the first follow‐up and from 0.801 to 0.808 at the second follow‐up. As the number of patients increased, the performance of the three models improved.

## 4 DISCUSSION

The development and validation of the Doylestown algorithm and aMAP score in predicting HCC were performed using clinical variables from case‐control or nested case‐control studies.^15^, ^16^ Furthermore, Wang et al.^18^ developed a secondary Doylestown Plus algorithm that incorporated cosylated kininogen as a marker to improve the detection of HCC. Yamashita et al.^19^ investigated the clinical utility of the aMAP score for predicting HCC occurrence and the incidence‐free rate after a sustained virologic response in chronic hepatitis C. The current study was a retrospective, longitudinal study applying the AFP, Doylestown algorithm, and aMAP score to consecutive time points for patients with liver cirrhosis to compare the performance of the algorithm in detecting HCC. Additionally, to our knowledge, this study also investigated the application of the Doylestown algorithm to the Chinese population for the first time.

Among the three evaluation models, the specificity of the Doylestown algorithm was similar or slightly less than AFP, but the Doylestown algorithm showed the best performance, and the aMAP score performance was relatively poor. The Doylestown algorithm, by combining AFP with other clinical values, increased the detection rate of HCC when compared to AFP alone in all of the tested time points. In the 521 liver cirrhosis patients with triple follow‐up, using a fixed cutoff of 20 ng/mL for AFP and 0.5 output units for the Doylestown algorithm resulted in a 7.4% increased biomarker performance at the closest time point to HCC diagnosis (0–6 month prior), a 21% increase 6–12 months before diagnosis, and a 13% increase 12–18 months before diagnosis. The Doylestown algorithm demonstrated better performance for liver cirrhosis patients, and it was also suitable for the Chinese population. However, the overall Doylestown algorithm value for the Chinese population was lower than that of the Caucasian population. For example, 20 patients diagnosed with HCC had an average Doylestown algorithm of 0.49 0–6 months before diagnosis, and 17 patients diagnosed with HCC had an average Doylestown algorithm of 0.42 6–12 months before diagnosis. In comparison, studies have shown that the average Doylestown algorithm for Caucasian patients diagnosed with HCC 12 months before the diagnosis is 0.58.^20^ Therefore, we believe that the Doylestown algorithm is also suitable for Chinese patients with liver cirrhosis, but the cutoff value needed to be adjusted before application.

The aMAP score demonstrated the highest sensitivity in this analysis, but it introduced a very high number of false‐positive cases. One reason for this result could be because most of the aMAP score training sets were hepatitis B patients, and the performance of the model was not as favorable as that of the Doylestown algorithm for patients with liver cirrhosis. Among patients who developed HCC, when comparing a cutoff of 50 for the aMAP score to AFP at 20 ng/mL, the aMAP score advanced the average time for early diagnosis of HCC from 161 days to 299 days (Table S2). The number of people who were diagnosed in advance by AFP was the least, with only 5 patients. In contrast, the number of patients who were diagnosed in advance by aMAP was 18 (the highest). The aMAP score performance at the second follow‐up (6–12 months prior) was superior to that of the other two time periods. Compared with the Doylestown algorithm, the aMAP score may be more suitable for early screening of liver cancer (6–12 months in advance). Although the false‐positive rate was relatively high, this model could be fit for preliminary screening of liver cancer (to reduce missed diagnosis) and then be combined with other screening methods (to reduce misdiagnosis) to further confirm the suspected liver cancer patients.

A limitation of the current study was the small sample size and short follow‐up time. For example, clinical data were only available from 20 patients at a time of 0–6 months before HCC diagnosis. If restricted to samples within 12–18 months of HCC diagnosis, there were only 5 HCC patients. A larger study with time points up to 3–5 years prior to HCC diagnosis should be performed to fully demonstrate the true benefit of using this algorithm for HCC surveillance in the Chinese population. In addition, our study was conducted at a single center with a limited number of liver disease patients. It is necessary to verify the Doylestown algorithm through a multi‐center cooperative study. At the same time, we must recognize that the Doylestown algorithm and the aMAP score can detect HCC patients in advance compared with traditional imaging screening methods, but the sensitivity and specificity of the model should be improved.

This study demonstrated that the Doylestown algorithm, by using readily available clinical parameters, is superior to AFP alone in accurately predicting the development of HCC among patients in the Chinese population with liver cirrhosis. While this algorithm is practical and easy to adopt in routine clinical use, it is important to emphasize that the current algorithm can be further complemented and improved by combining with novel biomarkers such as DNA, RNA, proteins, exosomes, or epigenetic markers to achieve early detection of HCC in the future.

## 5 CONCLUSIONS

In summary, our study results highlight the potential of novel biomarker panels, including the Doylestown algorithm and aMAP score, to improve early HCC detection. The high accuracy of these biomarker panels is likely related to the inclusion of multiple biomarkers and demographics associated with higher HCC risk. Blood‐based biomarkers also have high patient acceptance and are easy to implement in clinical practice. The performance of the Doylestown algorithm was superior to that of AFP and aMAP score, and the algorithm was also applicable to the Chinese population. The aMAP score demonstrated very high sensitivity and was suitable for preliminary HCC screening, but it is necessary to combine with other screening methods to further identify suspected HCC patients.

## CONFLICTS OF INTEREST

Linhong Li and Juhong Zhou are employees of Oriomics Biotech Inc. No other disclosures were reported. The authors have no conflicts of interest to disclose.

## CONSENT FOR PUBLICATION

Not applicable.

## Supporting information

Table S1‐S2

## Data Availability

The data supporting the findings of this study are available within the article and its supplementary materials or are available from the corresponding authors upon reasonable request.

## References

[1] Sung H , Ferlay J , Siegel RL , et al. Global cancer statistics 2020: GLOBOCAN estimates of incidence and mortality worldwide for 36 Cancers in 185 countries. CA: A Cancer J Clin. 2021;71(3):209–249. doi:10.3322/caac.21660 33538338

[2] Zhou J , Sun H , Wang Z , et al. Guidelines for the diagnosis and treatment of hepatocellular carcinoma. Liver Cancer. 2020;9:682‐720. 33442540 10.1159/000509424 PMC7768108

[3] Sarveazad A , Agah S , Babahajian A , et al. Predictors of 5 year survival rate in hepatocellular carcinoma patients. J Res Med Sci. 2019;24(1):86. doi:10.4103/jrms.JRMS_1017_18 31741658 PMC6856560

[4] Borie F , Bouvier AM , Herrero A , et al. Treatment and prognosis of hepatocellular carcinoma: a population based study in France. J Surg Oncol. 2008;98(7):505–509. doi:10.1002/jso.21159 18932235

[5] El‐Serag HB . Hepatocellular carcinoma. N Engl J Med. 2011;365(12):1118–1127. 21992124 10.1056/NEJMra1001683

[6] Bruix J , Sherman M . Management of hepatocellular carcinoma. Hepatology. 2005;42(5):1208–1236. doi:10.1002/hep.20933 16250051

[7] Ioannou GN , Perkins JD , Carithers RL Jr . Liver transplantation for hepatocellular carcinoma: impact of the MELD allocation system and predictors of survival. Gastroenterology. 2008;134(5):1342–1351. doi:10.1053/j.gastro.2008.02.013 18471511

[8] Wang FS , Fan JG , Zhang Z , et al. The global burden of liver disease: the major impact of China. Hepatology. 2014;60(6):2099–2108. doi:10.1002/hep.27406 25164003 PMC4867229

[9] Professional Committee for Prevention and Control of Hepatobiliary and Pancreatic Diseases of Chinese Preventive Medicine Association , Professional Committee for Hepatology, Chinese Research Hospital Association , Chinese Society of Hepatology, Chinese Medical Association . Guideline for stratified screening and surveillance of primary liver cancer(2020 Edition). Zhonghua Gan Zang Bing Za Zhi. 2021;43(1):60–77. doi: 10.3760/cma.j.cn112152-20201109-00970 33541021

[10] Alavekios DA , Dionysian E , Sodl J , et al. Longitudinal analysis of effects of operator experience on accuracy for ultrasound detection of supraspinatus tears. J Shoulder Elbow Surg. 2013;22(3):375–380. doi:10.1016/j.jse.2012.09.017 23312821

[11] Bril F , Ortiz‐Lopez C , Lomonaco R , et al. Clinical value of liver ultrasound for the diagnosis of nonalcoholic fatty liver disease in overweight and obese patients. Liver Int. 2015;35:2139‐2146. doi:10.1111/liv.12840 25847730

[12] Gupta S , Bent S , Kohlwes JJAoim . Test characteristics of α‐fetoprotein for detecting hepatocellular carcinoma in patients with hepatitis C. Ann Int Med. 2003;139:46‐50. 12834318 10.7326/0003-4819-139-1-200307010-00012

[13] Ismail MM , Morsi HK , Abdulateef NA , et al. Evaluation of prothrombin induced by vitamin K absence, macrophage migration inhibitory factor and Golgi protein‐73 versus alpha fetoprotein for hepatocellular carcinoma diagnosis and surveillance. Scand J Clin Lab Investig. 2017;77(3):175–183. doi:10.1080/00365513.2017.1286684 28276727

[14] Wong GL , Chan HL , Tse YK , et al. On‐treatment alpha‐fetoprotein is a specific tumor marker for hepatocellular carcinoma in patients with chronic hepatitis B receiving entecavir. Hepatology. 2014;59(3):986–995. doi:10.1002/hep.26739 24123097

[15] Wang M , Devarajan K , Singal AG , et al. The Doylestown algorithm: a test to improve the performance of AFP in the detection of hepatocellular carcinoma. Cancer Prev Res. 2016;9(2):172–179. doi:10.1158/1940-6207.CAPR-15-0186 PMC4740237 26712941

[16] Fan R , Papatheodoridis G , Sun J , et al. aMAP risk score predicts hepatocellular carcinoma development in patients with chronic hepatitis. J Hepatol. 2020;73(6):1368–1378. doi:10.1016/j.jhep.2020.07.025 32707225

[17] Llovet JM , Brú C , Bruix J . Prognosis of hepatocellular carcinoma: the BCLC staging classification. Sem Liver Dis. 1999;19(03):329–338. 10.1055/s-2007-1007122 10518312

[18] Wang M , Sanda M , Comunale MA , et al. Changes in the glycosylation of kininogen and the development of a kininogen‐based algorithm for the early detection of HCC. Cancer Epidemiol Biomarkers Prev. 2017;26(5):795–803. doi:10.1158/1055-9965.EPI-16-0974 28223431 PMC5759760

[19] Yamashita Y , Joshita S , Sugiura A , et al. aMAP score prediction of hepatocellular carcinoma occurrence and incidence‐free rate after a sustained virologic response in chronic hepatitis C. Hepatol Res. 2021;51(9):933–942. doi:10.1111/hepr.13689 34216422

[20] Mehta AS , Lau DT , Wang M , et al. Application of the Doylestown algorithm for the early detection of hepatocellular carcinoma. PLoS One. 2018;13(8):e0203149. doi:10.1371/journal.pone.0203149 30169533 PMC6118370

